# GABARAPL1 tumor suppressive function is independent of its conjugation to autophagosomes in MCF-7 breast cancer cells

**DOI:** 10.18632/oncotarget.19639

**Published:** 2017-07-27

**Authors:** Laura Poillet-Perez, Marine Jacquet, Eric Hervouet, Thierry Gauthier, Annick Fraichard, Christophe Borg, Jean-René Pallandre, Bruno J. Gonzalez, Yasmina Ramdani, Michaël Boyer-Guittaut, Régis Delage-Mourroux, Gilles Despouy

**Affiliations:** ^1^ Unité Mixte de Recherche, Interactions Hôte-Greffon-Tumeur, Ingénierie Cellulaire et Génique, Université Bourgogne Franche-Comté, Besançon, France; ^2^ Microvascular Endothelium and Neonatal Brain Lesions, Université de Normandie, UFR de Médecine et de Pharmacie, Rouen, France; ^3^ Rutgers Cancer Institute of New Jersey, New Brunswick, New Jersey, USA

**Keywords:** breast cancer, GABARAPL1, LC3, MCF-7, Autophagy

## Abstract

The GABARAPL1 protein belongs to the ATG8 family whose members are involved in autophagy. Our laboratory previously demonstrated that GABARAPL1 associates with autophagic vesicles, regulates autophagic flux and acts as a tumor suppressor protein in breast cancer. In this study, we aimed to determine whether GABARAPL1 conjugation to autophagosomes is necessary for its tumor suppressive functions using the MCF-7 breast cancer cell line overexpressing GABARAPL1 or a G116A mutant, which is unable to be lipidated and associated to autophagosomes. We show that the G116A mutation impaired GABARAPL1 function in autophagosome/lysosome fusion and inhibited lysosome activity but did not alter MTOR and ULK1 activities or tumor growth *in vivo*. Our results demonstrate for the first time that GABARAPL1 plays different regulatory functions during early and late stages of autophagy, independently or not of its conjugation to autophagosomes, but its tumor suppressive function appeared to be independent of its conjugation to autophagic vesicles.

## INTRODUCTION

Macroautophagy (hereafter called autophagy) is a cellular degradation process in which damaged proteins, organelles and other cytoplasmic constituents are degraded and recycled to provide nutrients and energy [1, 2]. This process is characterized by the engulfment of portions of the cytosol, soluble proteins and/or organelles into a unique double-membrane structure called the phagophore. The phagophore elongates and closes to generate a double-membrane organelle called the autophagosome which then fuses with the lysosome to form the autophagolysosome, leading to the degradation of its content by lysosomal hydrolases [3, 4]. This mechanism occurs at low basal levels to maintain cellular homeostasis but can be induced by different stresses such as hypoxia or nutrient starvation to allow cell survival. These stresses induce different signaling pathways involving MTORC1 (mechanistic target of rapamycin complex), an autophagy inhibitor, or AMPK (AMP-activated protein kinase), an inducer of autophagy. MTORC1 and AMPK then phosphorylate the same protein called ULK1 (unc-51 like autophagy activating kinase 1), the yeast homolog of Atg1, to modulate autophagy following nutrient or energy starvation. During nutrient-rich conditions, MTORC1 associates with the ULK1 complex and inhibits autophagy through the phosphorylation of ULK1 at Ser 757. Nutrient starvation leads to the inhibition of MTORC1, activation of ULK1 and subsequent induction of autophagy [5, 6]. During nutrient or energy starvation, AMPK can inhibit MTORC1 and activate ULK1 through phosphorylation at Ser 317, 555 and 777 leading to the induction of autophagy [7–9].

The autophagy process is mediated by more than 30 autophagy-related (ATG) proteins. Among these proteins, GABARAPL1/GEC1 (GABA_A_ receptor-associated protein-like 1/Glandular epithelial cells 1), which was first described by our group as an estrogen regulated gene, belongs to the ATG8 family [10], composed of 2 subfamilies the MAP-LC3 (microtubule-associated light chain-3) subfamily and the GABARAP subfamily. The latter includes GABARAPL1, GABARAP and GABARAPL2/GATE-16 (GABARAP like-2 protein/Golgi-associated ATPase enhancer of 16 kDa), which share 87% and 61% identity with GABARAPL1, respectively [11–13]. GABARAPL1 is composed of 117 amino acids and is involved in protein intracellular transport due to its interaction with the GABA_A_ receptor, the κ opioid receptor and TUBULIN [14, 15]. GABARAPL1 also interacts with HSP-90 (Heat shock protein 90) which prevents its degradation by the proteasome [16].

The members of the ATG8 family possess a conserved C-terminal glycine at position 116 (GABARAP family) or 120 (LC3 family) which is essential for their conjugation to autophagosomes and their role in autophagy [17]. Indeed, during autophagy, GABARAPL1, like the other members of ATG8 family, is cleaved by the protease ATG4B which exposes its C-terminal glycine, and give the cytosolic mature form called GABARAPL1-I [18]. During phagophore elongation, this cytosolic form is linked to phosphatidylethanolamine, by the ATG7 (E1-like) and ATG3 (E2-like) enzymes, to give rise to the membrane-associated form, called GABARAPL1-II [19–23].

Despite their homology, GABARAP and LC3 subfamily members are suspected to be involved at different stages of the autophagic process. Indeed, the LC3 subfamily is thought to be necessary for the elongation of the phagophore whereas the GABARAP subfamily has been suggested to be necessary for the maturation of the autophagosome [24]. In a previous study, we demonstrated that a cellular knock-down of GABARAPL1 decreased autophagic flux and lysosome number in the breast cancer cell line MDA-MB-436 [25]. This knock-down also led to an increase in glutathione and ATP level, basal respiration and accumulation of damaged mitochondria, suggesting a function of GABARAPL1 in mitochondrial homeostasis and metabolic reprogramming. Moreover, GABARAPL1 has been shown to interact with AMPK (unpublished data) and ULK1 suggesting a potential involvement of this protein during the early stages of autophagy, as well [26, 27].

The ATG8 family is also involved in “selective autophagy” which targets defined cargos for degradation [28]. Cargo adaptor proteins interact with ATG8 proteins via their LIR motif (LC3-interacting region) to deliver ubiquitinated proteins or organelles to the autophagosomes for their degradation [29, 30]. For example, GABARAPL1 interacts with the cargo receptors SQSTM1 (sequestosome 1) or NBR1 (neighbor of BRCA1 gene 1) to induce the degradation of ubiquitinated protein aggregates (cargos) and with NIX to activate the degradation of damaged mitochondria during the selective process called mitophagy [31–33].

Deregulation of autophagy is thought to be involved in various diseases including cancer [34]. However, autophagy presents a double edge-sword in cancer since it can act as a tumor-suppressing or a tumor-promoting process depending on tumor type and stage [35–37]. During the early stages of tumorigenesis, autophagy acts as a tumor suppressor mechanism by limiting DNA damage, chromosome instability, oxidative stress and inflammation which are oncogenic stimuli [38, 39]. Moreover, the expression of proteins involved in autophagy such as BECN1 (BECLIN-1), ATG5, UVRAG (UV radiation resistance-associated gene), GABARAP and LC3 has been described to be reduced or lost in several types of cancers [40–44]. On the contrary, during the later stages of tumorigenesis, some cancer cells present elevated autophagy levels allowing them to survive against metabolic stress. Indeed, the microenvironment of cancer cells presents reduced levels of nutrients, oxygen and growth factors leading to an altered metabolism and an impairment of ATP production [45, 46].

Several studies have highlighted a role of GABARAPL1 as a tumor suppressor protein. We have previously shown in our laboratory that cancer cell lines present a reduced *GABARAPL1* expression compared to normal cells and that a high *GABARAPL1* expression is correlated with a good prognosis in breast cancer patients [47, 48]. Moreover, we have demonstrated that GABARAPL1 overexpression inhibits cell proliferation, colony formation and invasion of breast cancer cells *in vitro* [25, 47]. These results are consistent with those recently demonstrating that GABARAPL1 expression is decreased in hepatocellular carcinoma (HCC) compared to adjacent liver tissue and that GABARAPL1 inhibits cell growth of HCC cancer cell lines [49]. It has also been shown that GABARAPL1 overexpression inhibits tumor growth *in vivo* and mediate the degradation of DVL2 (Dishevelled 2) through selective autophagy leading to the inhibition of the Wnt pathway whose deregulation has been described to be involved in various diseases such as cancer [50].

Given the function of GABARAPL1 in autophagy and cancer, the purpose of our study was to: i) study the role of GABARAPL1 during early and late stages of autophagy and, ii) determine the involvement of GABARAPL1 conjugation to autophagosomes in its tumor suppressive function. To do so, we used the breast cancer cell line MCF-7 overexpressing GABARAPL1 or GABARAPL1 G116A mutant protein in which the essential C-terminal glycine at position 116 has been replaced by an alanine.

## RESULTS

### The G116A mutation impaired the conjugation of GABARAPL1 to phospholipids and its recruitment to autophagosomes

**Figure 1:**
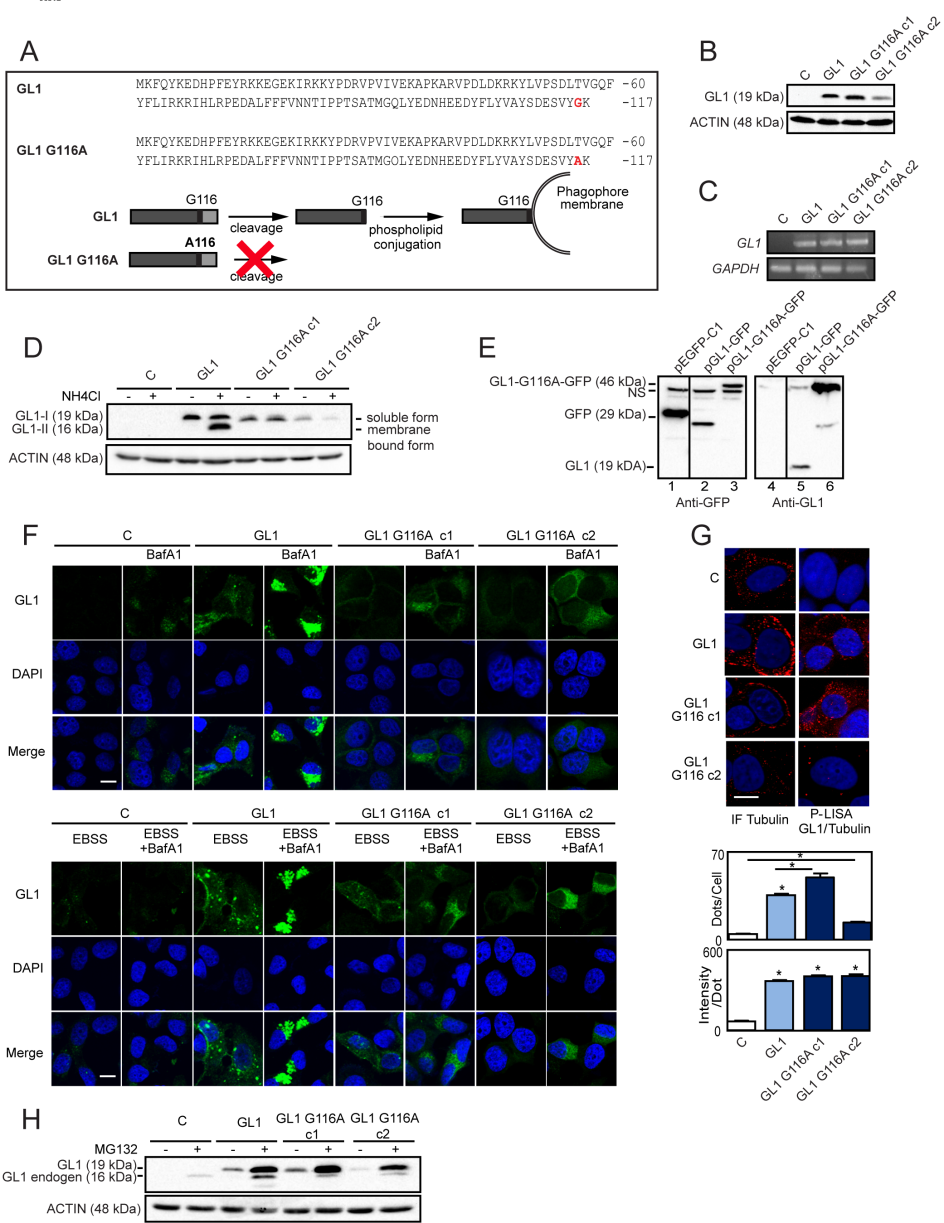
Characterization of MCF-7 overexpressing GABARAPL1 or GABARAPL1 G116A **A.** Alignment of the amino acid sequences of GABARAPL1 and GABARAPL1 G116A (Top). Schema representing the cleavage and lipidation of GABARAPL1 during autophagy (Bottom). **B.** Western blotting analysis of GABARAPL1 in MCF-7 C, GABARAPL1 and GABARAPL1 G116A cells. Data are representative of three independent experiments. **C.** qRT-PCR analysis of GABARAPL1 mRNA expression. Representative data of two independent experiments performed in duplicate are shown. **D.** Western blotting analysis of GABARAPL1 in MCF-7 C, GABARAPL1 and GABARAPL1 G116A cells cultured in medium with or without 50 mM NH_4_Cl for 2h. Data are representative of three independent experiments. **E.** Western blotting analysis of GFP and GABARAPL1 in MCF-7 cells transfected with the pGFP, pGABARAPL1-GFP and pGABARAPL1-G116A-GFP vectors. Data are representative of three independent experiments. **F.** Immunofluorescence analysis of GABARAPL1 in MCF-7 C, GABARAPL1 and GABARAPL1 G116A cells cultured in medium or EBSS with or without 100 nM BafA1 for 8 h. A representative image of two independent experiments performed in duplicate is shown. Scale bar represents 10 μm. **G.** P-LISA signals analysis of TUBULIN/GABARAPL1 interaction (red) and nuclei (blue) in MCF-7 C, GABARAPL1 and GABARAPL1 G116A cells. A representative image of three independent experiments is shown. The number of red dots and the intensity per dots were counted using the Blobfinder software. 200 cells were randomly selected in 5 fields. Data are means ± S.E.M. *P <0.05 compared to the control. Scale bar represents 5 μm. (H) Western blotting analysis of GABARAPL1 in MCF-7 C, GABARAPL1 and GABARAPL1 G116A cells cultured in medium with or without 2 μM MG132 for 16h. Data are representative of three independent experiments.

In order to determine the importance of the GABARAPL1 conjugation to autophagosomes on its tumor suppressive function, we designed MCF-7 breast cancer cell lines overexpressing either Flag:GABARAPL1:6His (GABARAPL1) or Flag:GABARAPL1-G116A:6His mutant (clone 1 and clone 2 ; GABARAPL1 G116A c1 and c2) (Figure 1A). First, we analyzed GABARAPL1 protein and mRNA expression levels in our cell models. As expected, GABARAPL1 and GABARAPL1 G116A expression were detected in MCF-7 GABARAPL1, GABARAPL1 G116A c1 and c2 cells but not in control cells transfected with the empty vector (Figures 1B-1C). Interestingly, we noted that MCF-7 GABARAPL1 G116A c1 cells showed a GABARAPL1 protein expression similar to the one observed in MCF-7 GABARAPL1 cells whereas MCF-7 GABARAPL1 G116A c2 cells presented a lower GABARAPL1 protein expression. We next wanted to check whether overexpression of GABARAPL1 modified the expression of its homologue GABARAP using an antibody which detects both proteins. Overexpression of GABARAPL1 or GABARAPL1 G116A in MCF-7 cells did not modify the expression of its homologue, GABARAP (Supplementary Figure S1A).

Our laboratory has previously reported that, during autophagy, GABARAPL1 needs to be cleaved, depending on its C-terminal glycine, before being associated to autophagic vesicles in HEK-293 cells [23]. We therefore wanted to know whether the G116A mutation impaired lipidation of GABARAPL1 and its localization to autophagosomes in MCF-7 cells. To do so, we studied GABARAPL1 expression in our different cell models following treatment with NH_4_Cl, a lysosomal activity inhibitor, which led to the accumulation of autophagosomes and the lipidated form of GABARAPL1 called GABARAPL1-II [23]. Without treatment, only the mature soluble GABARAPL1-I form (19 kDa) was detected in MCF-7 GABARAPL1 or GABARAPL1 G116A c1 and c2 cells (Figure 1D). As expected, in MCF-7 GABARAPL1 cells, NH_4_Cl treatment led to the appearance of the GABARAPL1-II form (16 kDa) but this treatment had no effect on MCF-7 GABARAPL1 G116A c1 and c2 cells. These results clearly demonstrate that the G116A mutation impaired the lipidation of GABARAPL1. In order to confirm that GABARAPL1 G116A cannot be cleaved, we transfected MCF-7 cells with three different vectors coding either GFP, GFP fused to the C-terminus of GABARAPL1 (GABARAPL1-GFP) or to the C-terminus of GABARAPL1 G116A (GABARAPL1-G116A-GFP) (Figure 1E). When GABARAPL1-GFP is expressed the anti-GFP antibody recognized the truncated GFP protein due to the cleavage of GABARAPL1 at G116 (lane 2). The difference in size between the native GFP (lane 1) and the cleaved GFP (lane 2) was probably due to a supplementary linker sequence added in the EGFP-C1 commercial vector. These results were confirmed by probing with the anti-GABARAPL1 antibody which detected GABARAPL1 alone (lane 5). When GABARAPL1-G116A-GFP was expressed, we did not observe the truncated form of GFP (lane 3) or GABARAPL1 (lane 6). These results confirmed that the G116A mutation impaired the cleavage of the GABARAPL1 protein in the MCF7 cells. Since GABARAP is also cleaved during autophagy to give the membrane-associated form called GABARAP-II, we wanted to verify that overexpression of GABARAPL1 or GABARAPL1 G116A did not alter the lipidation of GABARAP. As expected, NH_4_Cl led to an increase in the GABARAP-II form in MCF-7 C, GABARAPL1, GABARAPL1 G116A c1 and c2 cells (Supplementary Figure S1B). However, this increase appeared similar in our different cell lines confirming that overexpression of GABARAPL1 or GABARAPL1 G116A did not modify expression or lipidation of the GABARAP protein.

Next, we studied the cellular localization of GABARAPL1 and GABARAPL1 G116A after treatment with BafilomycinA1 (BafA1), an inhibitor of autophagosome/lysosome fusion. Without treatment, MCF-7 GABARAPL1 or GABARAPL1 G116A cells presented a diffuse GABARAPL1 staining in the cytoplasm (Figure 1F top panel). As expected, the treatment with BafA1 led to an accumulation of GABARAPL1 puncta around the nucleus in MCF-7 GABARAPL1 cells, indicating a relocalization of GABARAPL1 to autophagosomes. On the contrary, treatment of MCF-7 GABARAPL1 G116A cells with BafA1 did not lead to any accumulation of GABARAPL1 puncta, confirming that GABARAPL1 G116A was unable to conjugate to autophagic vesicles (Figure 1F top panel). Similar results were obtained after treatment with EBSS, an inducer of autophagy, in the presence or absence of BafA1 (Figure 1F bottom panel). Indeed, treatment with EBSS increased the intensity of GABARAPL1 staining and GABARAPL1 puncta in MCF-7 GABARAPL1 but only increased the intensity of GABARAPL1 staining in GABARAPL1 G116A c1 and c2 cells. As shown above, addition of BafA1 led to an accumulation of GABARAPL1 puncta in MCF-7 GABARAPL1 cells but not in MCF-7 GABARAPL1 G116A c1 and c2 cells.

In order to study the effect of the G116A mutation on other functions of GABARAPL1 (those suggested to be independent of its conjugation to autophagosomes), we studied the impact of this mutation on the interaction of GABARAPL1 with TUBULIN and the degradation of GABARAPL1 by the proteasome [14, 16]. Firstly, we studied the interaction between GABARAPL1 and TUBULIN using the P-LISA protocol (Proximity Ligation In Situ Assay), which allows the quantification of stable and transient interactions of endogenous proteins *in situ* [51]. Our experiments showed that GABARAPL1 G116A exhibited a similar level of interaction with TUBULIN compared to GABARAPL1 suggesting that the G116A mutation did not impair GABARAPL1/TUBULIN interaction (Figure 1G). Next, we studied endogenous or exogenous GABARAPL1 expression [Flag:GABARAPL1:6His and Flag:GABARAPL1-G116A:6His expression (19 kDa) or GABARAPL1 expression (16 kDa)] in our different cell models after treatment with MG132, a proteasome inhibitor (Figure 1H). As previously described, MG132 treatment led to an increase in both exogenous and endogenous GABARAPL1 expression levels in MCF-7 GABARAPL1 cells. Similar results were obtained in MCF-7 GABARAPL1 G116A cells, suggesting that the G116A mutation did not impair its degradation by the proteasome. On the contrary, MG132 treatment did not modify GABARAP expression levels in MCF-7 C, GABARAPL1, GABARAPL1 G116A c1 and c2 cells (Supplementary Figure S1C). These results therefore suggest that the G116A mutation specifically alters GABARAPL1 conjugation to autophagosomes without affecting other functions of this protein.

### The G116A mutation impaired the function of GABARAPL1 during induced but not basal autophagy

**Figure 2:**
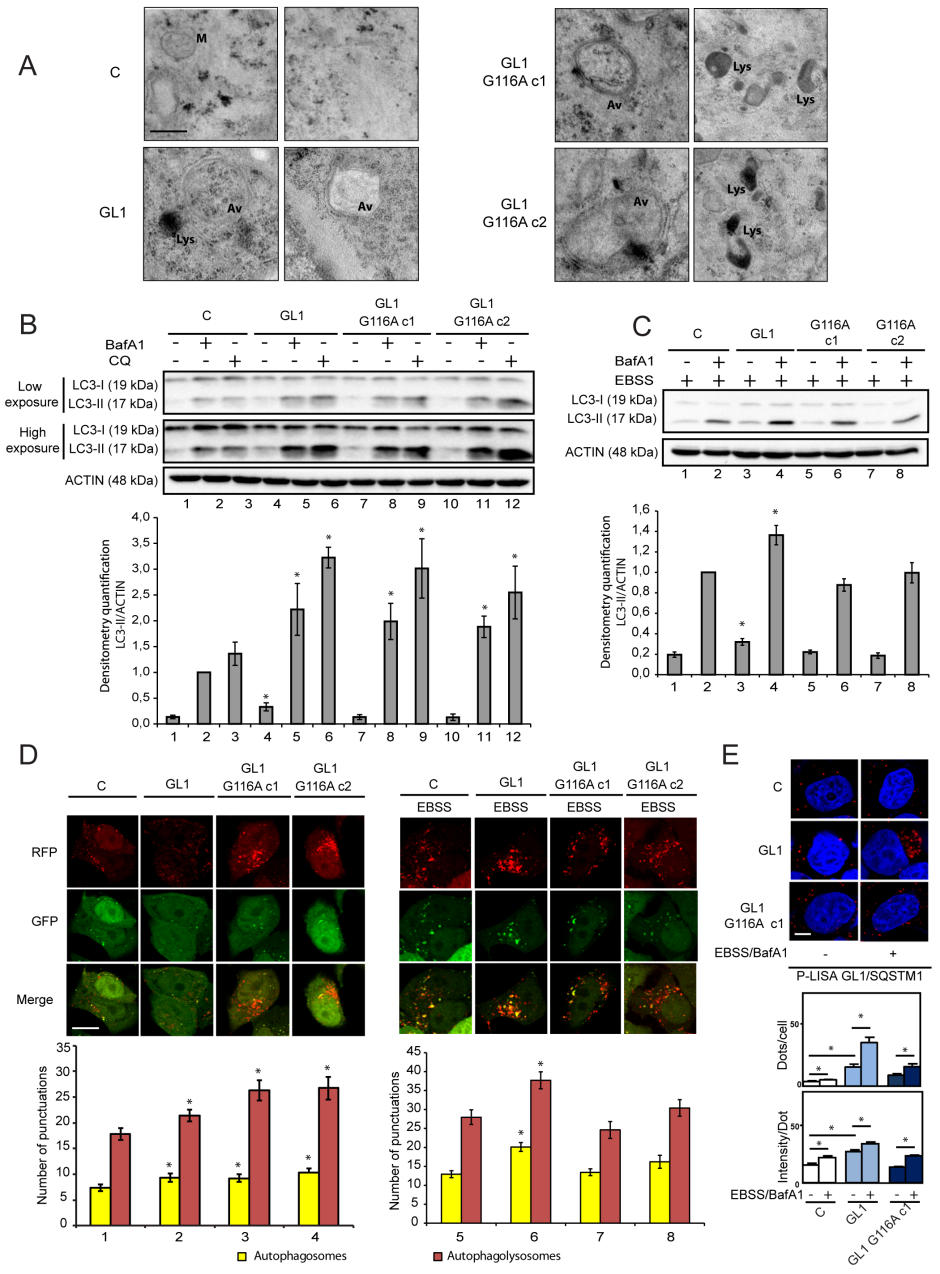
The G116A mutation impaired the effect of GABARAPL1 on induced but not basal autophagy **A.** Electron microscopy of MCF-7 C, GABARAPL1, GABARAPL1 G116A cells. Av: Autophagic vesicles; Lys: Lysosomes and M: Mitochondria. Scale bar represents 0.25 μm. A representative image of 60 pictures for each cell lines is shown. **B.** Western blotting analysis of LC3 in MCF-7 C, GABARAPL1 and GABARAPL1 G116A cells cultured in medium with or without 100 nM BafA1 or 40 μM Chloroquine (CQ) for 2h. A representative image of ten independent experiments is shown. LC3-II levels were quantified using the Image Lab software. Data are means ± S.E.M. of ten independent experiments. *P <0.05 compared to the associated control. **C.** Western blotting analysis of LC3 in MCF-7 C, GABARAPL1 and GABARAPL1 G116A cells cultured in EBSS for 4h with or without 100 nM BafA1 for 2h. A representative image of ten independent experiments is shown. LC3-II levels were quantified using the Image Lab. Data are means ± S.E.M. of ten independent experiments. *P <0.05 compared to the associated control. **D.** GFP-RFP-LC3 puncta analysis in MCF-7 C, GABARAPL1 and GABARAPL1 G116A cells transfected with the ptf-LC3 vector and cultured in medium or EBSS. Each picture is representative of a typical cell staining observed in 20 fields chosen at random. Red and yellow puncta were counted using the ImageJ software (Green and Red puncta colocalization tool). In each group, 20 cells were randomly selected. Data are means ± S.E.M. of three independent experiments. *P <0.05 compared to the control. Scale bar represents 10 μm. **E.** P-LISA signals analysis of SQSTM1/GABARAPL1 interaction (red) and nuclei (blue) in MCF-7 C, GABARAPL1 and GABARAPL1 G116A cells cultured in complete medium or EBSS for 4h with 100 nM BafA1 for 2h. A representative image of three independent experiments is shown. The number of red dots and the intensity per dots were counted using the Blobfinder software. 200 cells were randomly selected in 5 fields. Data are means ± S.E.M. *P <0.05 compared to the control. Scale bar represents 5 μm.

We then investigated, in a qualitative study, the effect of GABARAPL1 or GABARAPL1 G116A overexpression on autophagy by identifying autophagic vesicles using electron microscopy (Figure 2A). Overexpression of GABARAPL1 and GABARAPL1 G116A appears to lead to an increase in autophagic vesicles (Av) and lysosomes (Lys) compared to the control cells. Surprisingly, these results suggested that GABARAPL1 G116A, like GABARAPL1 might regulate autophagy induction.

Given these results, we next wanted to study the effect of GABARAPL1 or GABARAPL1 G116A expression on autophagic flux. Indeed, our laboratory has previously shown that knocking down GABARAPL1 decreased autophagic flux in MDA-MB-436 cells [25]. During autophagy, LC3B is cleaved and linked to a phospholipid at the autophagosome to form the membrane-associated form LC3-II. The amount of this protein is directly correlated with the number of autophagosomes [20]. We therefore analyzed the effect of GABARAPL1 or GABARAPL1 G116A overexpression on LC3-II levels. Without treatment, overexpression of GABARAPL1 led to an increase in LC3-II levels compared to the control cell lines. On the contrary, overexpression of GABARAPL1 G116A did not regulate the levels of LC3-II (Figure 2B, lanes 1-4-7-10). Since the amount of LC3-II is not directly related to the autophagic flux but represents the number of autophagosomes at a particular time point, an increase in LC3-II might either indicate an increase in autophagic flux or a decrease in autophagolysosome degradation. In order to study autophagic flux, we compared the amount of LC3-II in the presence or absence of BafA1 (Figure 2B, lanes 2-5-8-11) or Chloroquine (CQ) (Figure 2B, lanes 3-6-9-12), two inhibitors of autophagosome/lysosome fusion. Treatments with BafA1 and CQ led to a greater increase in LC3-II protein levels in MCF-7 GABARAPL1 and GABARAPL1 G116A cells compared to the control cells, suggesting an increased autophagic flux in these cells. These results suggested that GABARAPL1 might increase basal autophagic flux independently of its conjugation to autophagosomes. The effect of GABARAPL1 and GABARAPL1 G116A on induced autophagy, following treatment with EBSS, was then studied (Figure 2C). After treatment with EBSS, overexpression of GABARAPL1 led to an increase in LC3-II levels compared to the control cells whereas overexpression of GABARAPL1 G116A did not (Figure 2C, lanes 1-3-5-7). Combination of EBSS with BafA1 led to a greater increase in LC3-II levels in MCF-7 GABARAPL1 cells compared to the control cells (Figure 2C, lanes 2-4), suggesting increased autophagic flux. Interestingly, after treatment with EBSS and BafA1, MCF-7 GABARAPL1 G116A cells exhibited LC3-II levels similar to those observed in control cells (Figure 2C, lanes 2-6-8), suggesting that GABARAPL1 increased induced autophagic flux depending on its conjugation to autophagosomes.

In order to confirm these results, we used a double-tagged GFP-RFP-LC3 construct and studied the effect of GABARAPL1 or GABARAPL1 G116A overexpression on autophagosome and autophagolysosome numbers [52]. Since GFP fluorescence is sensitive to acidic and proteolytic conditions found in lysosomes but RFP fluorescence is not, this construct allows the discrimination of autophagosomes (RFP+/GFP+, yellow) and autophagolysosomes (RFP+/GFP-, red). Therefore, an increase in autophagic flux would result in a concomitant increase of red and yellow puncta. We used CQ as a negative control of this experiment. Indeed, the use of CQ led to an increase in yellow puncta but not red puncta specific of a blockade of autophagic flux (Supplementary Figure S2A). Without treatment, overexpression of GABARAPL1 and GABARAPL1 G116A increased the number of autophagosomes as well as autophagolysosomes compared to the levels observed in control cells (Figure 2D left panel). These results suggested that GABARAPL1 and GABARAPL1 G116A increased the basal autophagic flux. We next studied the number of autophagosomes and autophagolysosomes during induced autophagy. After treatment with EBSS, overexpression of GABARAPL1 led to a greater increase in autophagic flux, characterized by an increase in autophagosomes and autophagolysosomes number (Figure 2D right panel). On the contrary, after treatment with EBSS, overexpression of GABARAPL1 G116A led to a similar number of autophagosomes and autophagolysosomes compared to the one observed in control cells suggesting that GABARAPL1 increased induced autophagic flux depending on its conjugation to autophagosomes. Altogether, results obtained by western blotting and GFP-RFP-LC3 transfection suggested that GABARAPL1 seemed to present different functions in basal or induced autophagy, which are dependent or independent of its conjugation to autophagosomes.

It has been previously shown that GABARAPL1, like the other members of ATG8 family, interacts with SQSTM1 during selective autophagy [29]. In fact, SQSTM1 recognizes ubiquitinylated aggregates or organelles and then interacts with ATG8 proteins localized at the autophagosome membrane to direct their degradation. Moreover, it has been shown that the G120A mutation in LC3 or the G116A mutation in GABARAP or GABARAPL2/GATE-16 impaired their interaction with SQSTM1 [19, 21, 53]. Therefore we next wanted to confirm wether the G116A mutation also impaired the interaction of GABARAPL1 with SQSTM1. We therefore analyzed the interaction between SQSTM1 and GABARAPL1 in MCF-7 GABARAPL1 or GABARAPL1 G116A cells using P-LISA (Figure 2E). First, we observed that the overexpression of GABARAPL1 increased its interaction with SQTM1. As expected, treatment with EBSS and BafA1 led to an increased interaction of SQSTM1 with GABARAPL1. Without treatment, GABARAPL1 G116A did not interact with SQSTM1 since the signals observed were similar to the ones quantified for the interaction between endogenous GABARAPL1 and SQSTM1 in MCF-7 control cells. When MCF7 GABARAPL1 G116A cells were treated with EBSS and BafA1, we observed an increase in cellular dots but to a lower extent than the one quantified for GABARAPL1, suggesting that SQSTM1 interacts preferentially with GABARAPL1 when it is localized in autophagosomes. We confirmed these results by studying the colocalization between SQSTM1 and GABARAPL1 or GABARAPL1 G116A (Supplementary Figure S2B). Our results showed that, GABARAPL1 G116A can still interact with SQSTM1 but to a lesser extent than the interaction observed between GABARAPL1 and SQSTM1. Altogether, these results suggested that the G116A mutation, which prevented the conjugation of GABARAPL1 onto the autophagosomes, might impair its interaction with the cargo adaptor SQSTM1 and consequently its function in selective autophagy.

### The G116A mutation impaired GABARAPL1 functions during late stages of autophagy

**Figure 3:**
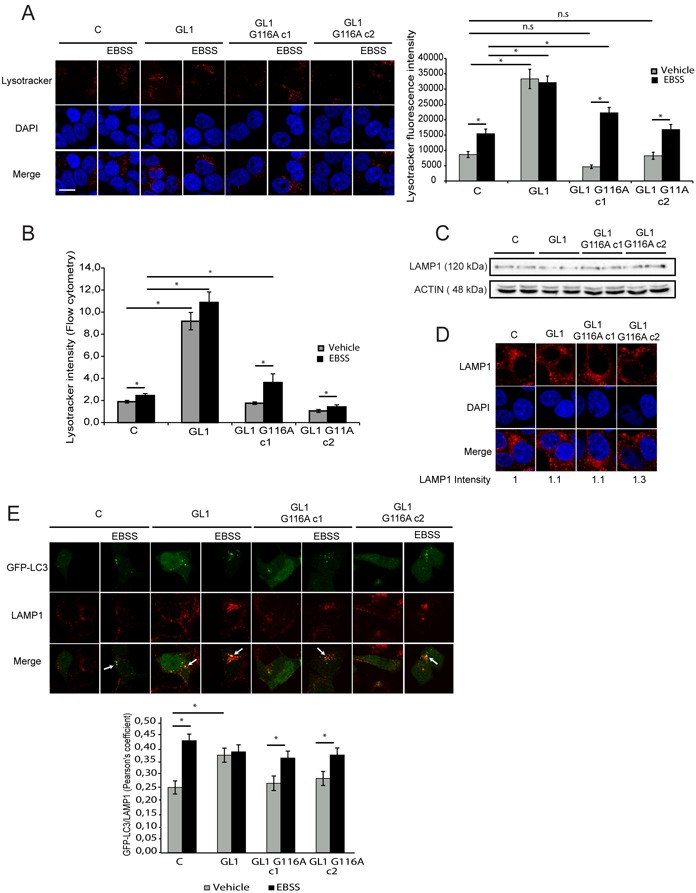
The G116A mutation impaired GABARAPL1 functions during late stages of autophagy **A.** Lysotracker staining in MCF-7 C, GABARAPL1 and GABARAPL1 G116A cultured in medium or EBSS for 4h observed with confocal microscope and quantified with the Blobfinder software. For each group, 100 cells were randomly selected. The data representative of three independent experiments are shown. Data are means ± S.E.M. *P <0.05 compared to the control. Scale bar represents 10 μm. **B.** Intensity of Lysotracker fluorescence analyzed by flow cytometry using the flowing software. Data are means ± S.E.M. of four independent experiments performed in duplicate. *P <0.05 compared to the control. **C.** Western blotting analysis of LAMP1 in MCF-7 C, GABARAPL1 and GABARAPL1 G116A cells. Data representative of three independent experiments performed in duplicate are shown **D.** Immunofluorescence analysis of LAMP1 in MCF-7 C, GABARAPL1 and GABARAPL1 G116A cells. A representative image of three independent experiments is shown. Scale bar represents 10 μm. **E.** Colocalization of LC3 and LAMP1 in MCF-7 C, GABARAPL1 and GABARAPL1 G116A cells transfected with the pGFP-LC3 vector and immunostained for LAMP1. Colocalization of the autophagosome marker GFP-LC3 and the lysosomal marker LAMP1 was analyzed using a confocal microscope and the Pearson's coefficient using coloc_2 (ImageJ software). For each group, 25 cells were randomly selected. The data representative of two independent experiments are shown. Data are means ± S.E.M. *P <0.05 compared to the control.

During the later stages of autophagy, a mature autophagosome fuses with a lysosome, a digestive organelle with an acidic lumen, to allow protein degradation and turnover. We previously reported that a knock-down of GABARAPL1 in MDA-MB-436 cells led to a decreased number of lysosomes compared to the control cells [25]. The effect of GABARAPL1 and GABARAPL1 G116A overexpression on lysosome acidification was then studied using Lysotracker, a fluorescent dye which specifically label acidic compartments including lysosomes. Confocal microscopy analysis showed that overexpression of GABARAPL1 led to an increase in Lysotracker fluorescence suggesting an increase in lysosomal acidification (Figure 3A). On the contrary, overexpression of GABARAPL1 G116A did not change the Lysotracker fluorescence levels compared to control cells. Treatment with EBSS led to an increase in Lysotracker fluorescence in control cells and cells overexpressing GABARAPL1 G116A. Interestingly, treatment with EBSS did not modify Lysotracker fluorescence in cells overexpressing GABARAPL1, probably because the levels in non-treated cells are already elevated. Furthermore, we did not detect any change in the percentage of Lysotracker-positive vesicules following overexpression of GABARAPL1 or GABARAPL1 G116A (Supplementary Figure S3A). These results suggested that GABARAPL1 increased the acidification of lysosome depending on its conjugation to autophagosomes. These results were confirmed by the quantification of Lysotracker fluorescence using a flow cytometer (Figure 3B). Since an increase in lysosomal acidification could be linked to an increase in lysosomal number, we then studied the effect of GABARAPL1 or GABARAPL1 G116A overexpression on LAMP1 (Lysosome associated membrane protein 1) protein expression, a lysosomal marker. Our results showed that overexpression of GABARAPL1 and GABARAPL1 G116A did not significantly modify the levels of LAMP1 compared to those observed in control cells (Figure 3C). This result was confirmed by the observation of LAMP1 immunostaining using confocal microscopy. Indeed, overexpression of GABARAPL1 or GABARAPL1 G116A did not modify the fluorescence intensity of LAMP1 (Figure 3D).

The lysosome is a digestive organelle with an acidic lumen containing hydrolases such as cathepsins which are involved in the intracellular degradation and turnover of proteins. We next studied the activity of CATHEPSIN B during basal and induced autophagy using MagicRed (MR) (Supplementary Figure S3B). Without EBSS treatment, overexpression of GABARAPL1 led to an increase in MR fluorescence whereas overexpression of GABARAPL1 G116A did not. Moreover, during EBSS treatment, overexpression of GABARAPL1 led to a further increase in MR fluorescence compared to the one observed in the other cell lines (Supplementary Figure S3B). These preliminary results could suggest that the increase in lysosomal acidification observed in MCF-7 GABARAPL1 cells led to an increase in CATHEPSIN B activity. An increased lysosomal acidification and activity could be due to an increased autophagosome/lysosome fusion [54]. In order to verify this hypothesis, we then studied the effect of GABARAPL1 or GABARAPL1 G116A overexpression on GFP-LC3/LAMP1 colocalization, an event correlated with autophagosome/lysosome fusion (Figure 3E). Overexpression of GABARAPL1 led to an increase in GFP-LC3/LAMP1 colocalization compared to control cells whereas overexpression of GABARAPL1 G116A did not. EBSS treatment led to an increase in GFP-LC3/LAMP1 colocalization in control cells and cells overexpressing GABARAPL1 G116A compared to non-treated cells. In cells overexpressing GABARAPL1, treatment with EBSS did not modify GFP-LC3/LAMP1 colocalization, probably because, as described for Lysotracker fluorescence, the levels in non-treated cells are already elevated. These results suggested that GABARAPL1 increased autophagosome/lysosome fusion depending on its conjugation to autophagosomes. In order to examine whether GABARAPL1 or GABARAPL1 G116A localized at the autophagolysosome, we studied colocalization between GABARAPL1 or GABARAPL1 G116A and LAMP1 (Supplementary Figure S2C). We observed that GABARAPL1 but not GABARAPL1 G116A colocalized with LAMP1 during autophagy, suggesting that the G116A mutation impaired the localization of GABARAPL1 to lysosomes, which is consistent with the fact that this protein could not be linked to autophagosomes anymore. These results suggested that GABARAPL1 might increase autophagosome/lysosome fusion depending on its conjugation to autophagosomes, which could then also explain the increased lysosomal acidification observed in MCF-7 GABARAPL1 cells.

### The G116A mutation did not impair GABARAPL1 function during early stages of autophagy

**Figure 4:**
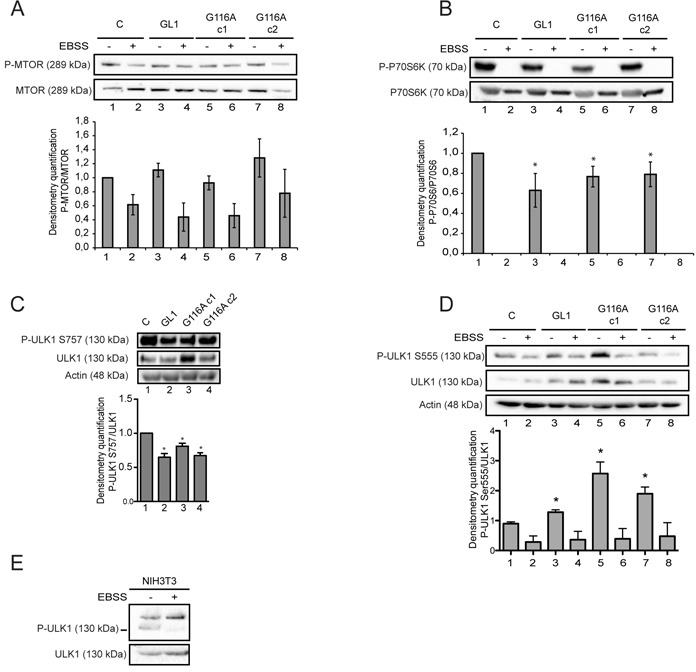
The G116A mutation did not impair GABARAPL1 functions during early stages of autophagy Western blotting analysis of MTOR phosphorylation **A.** P70S6 phosphorylation **B.** and ULK1 phosphorylation at Ser555 **D.** in MCF-7 C, GABARAPL1 and GABARAPL1 G116A cells cultured in medium or EBSS for 4h. Protein levels were quantified using the Image Lab. Representative image of four independent experiments is shown. Data are means ± S.E.M. of four independent experiments. *P <0.05 compared to the control. **C.** Western blotting analysis of ULK1 phosphorylation at Ser757 in MCF-7 C, GABARAPL1, and GABARAPL1 G116A cells. Protein levels were quantified using the Image Lab. Representative image of three independent experiments is shown. Data are means ± S.E.M. of three independent experiments. *P <0.05 compared to the control. **E.** Western blotting analysis of ULK1 phosphorylation at Ser555 in NIH3T3 cells cultured in medium or EBSS for 4h. Protein levels were quantified using the Image Lab. Representative image of two independent experiments is shown.

Since it has been previously shown that GABARAPL1 interacts with ULK1 [26, 27], a protein involved in initial events of autophagosome formation, we then studied the effect of GABARAPL1 and GABARAPL1 G116A overexpression during the induction of autophagy and particularly MTOR, a well-known ULK1 inhibitor and autophagy inhibitor [26, 27]. Without treatment overexpression of GABARAPL1 or GABARAPL1 G116A did not significantly modify MTOR phosphorylation (Figure 4A, lanes 1-3-5-7). Moreover, treatment with EBSS led to the same decrease in MTOR phosphorylation in MCF-7 GABARAPL1, GABARAPL1 G116A c1 or c2 cells (Figure 4A, lanes 2-4-6-8). We therefore studied the effect of GABARAPL1 and GABARAPL1 G116A overexpression on the phosphorylation of P70S6K (p70S6 kinase), a cellular MTOR target (Figure 4B). Without treatment, overexpression of GABARAPL1 and GABARAPL1 G116A led to a decreased phosphorylation of P70S6K compared to control cells suggesting that GABARAPL1 inhibited MTOR activity and consequently P70S6K phosphorylation independently of its conjugation to autophagosomes. Treatment with EBSS led to total loss of P70S6K phosphorylation in our cell lines (Figure 4B). Since ATG8 family members and more particularly GABARAP family proteins have been described to interact with ULK1 and allow its conjugation to autophagosomes [26], we wanted to analyze the effect of GABARAPL1 and GABARAPL1 G116A overexpression on the phosphorylation of ULK1 at Ser757, which is a direct target of MTOR and is associated with an inhibition of autophagy [7]. Overexpression of GABARAPL1 and GABARAPL1 G116A c1 led to a decreased phosphorylation of ULK1 at Ser757 (Figure 4C), confirming that GABARAPL1 indeed inhibited MTOR activity and ULK1 phosphorylation at Ser757 independently of its conjugation to the autophagosomes. We then examined another phosphorylation site of ULK1 at Ser555, a phosphorylation event which has been described to be necessary for autophagy induction [8]. Without treatment, overexpression of GABARAPL1 and GABARAPL1 G116A led to an increased phosphorylation of ULK1 at Ser555 (Figure 4D, lanes 1-3-5-7). It is interesting to note that overexpression of GABARAPL1 and GABARAPL1 G116A also increased total levels of the ULK1 protein. It has been previously shown in NIH3T3 cell lines that starvation induced by Krebs-Henseleit medium deprived of amino acids led to an increase in phosphorylation of ULK1 at Ser 555 [55]. On the contrary, in an MCF7 cells, treatment with EBSS led to a complete decrease in ULK1 phosphorylation. So, we repeated our experiment in NIH3T3 cells (Figure 4E) and demonstrated that treatment with EBSS also led to a decrease in Ser555 phosphorylation of ULK1 in NIH3T3 cell line. The use of different starvation medium may therefore explain these contradictory results.

Altogether, these results suggested that GABARAPL1 could regulate early induction autophagy steps independently of its conjugation to autophagosomes.

### The G116A mutation modified the function of GABARAPL1 for some cancer cell phenotypes *in vitro* but not tumor growth *in vivo*

**Figure 5:**
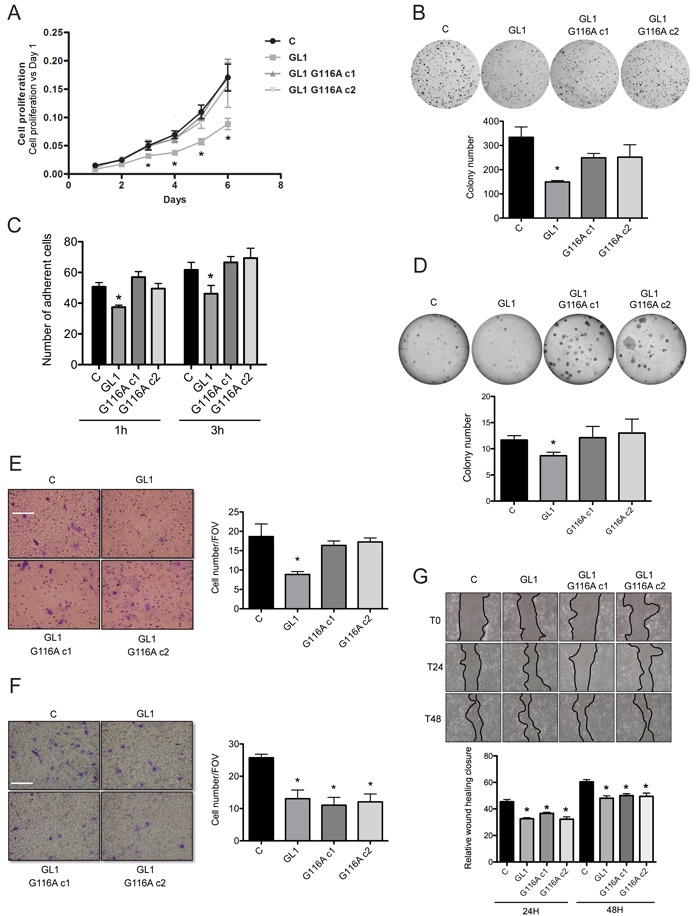
The G116A mutation modified the effect of GABARAPL1 on some cancer cell phenotypes *in vitro* **A.** Growth rate of MCF-7 C, GABARAPL1 and GABARAPL1 G116A cells using MTT assay. The data representative of three independent experiments performed in 24 replicates are shown. Data are means ± S.E.M of three independent experiments. *P <0.05 compared to the control. **B.** Clonogenic assay of MCF-7 C, GABARAPL1 and GABARAPL1 G116A cells. The colony numbers were evaluated by counting using Vision Capt software. The data representative of three independent experiments performed in duplicate are shown. Data are means ± S.E.M of three independent experiments. *P <0.05 compared to the control. **C.** Adhesion of MCF-7 C, GABARAPL1 and GABARAPL1 G116A cells. The data representative of three independent experiments performed in triplicate are shown. Data are means ± S.E.M of three independent experiments. *P <0.05 compared to the control. **D.** Colonies formation in soft agar of MCF-7 C, GABARAPL1 and GABARAPL1 G116A cells. Representative cell colonies in soft agar are shown. Data representative of three independent experiments performed in duplicate are shown. Data are means ± S.E.M of three independent experiements. *P <0.05 compared to the control. **E.** Invasion of MCF-7 C, GABARAPL1, GABARAPL1 G116A c1 and c2 cells in Boyden-modified chamber. A representative image of ten fields of view (FOV) of each membrane is shown. 10 FOV were randomly selected and the number of invasive cells was determined. Data representative of three independent experiments performed in duplicate are shown. Data are means ± S.E.M of three independent experiments. *P <0.05 compared to the control. Scale bar represents 10 μm. **F.** Migration of MCF-7 C, GABARAPL1 and GABARAPL1 G116A cells in Boyden-modified chamber. A representative image of ten fields of view (FOV) of each membrane is shown. 10 FOV were randomly selected and the number of migrative cells was determined. Data representative of three independent experiments performed in duplicate are shown. Data are means ± S.E.M of three independent experiments. *P <0.05 compared to the control. Scale bar represents 10 μm. **G.** Migration of MCF-7 C, GABARAPL1 and GABARAPL1 G116A cells using wound healing assay. Data representative of two independent experiments performed in 8 replicates are shown. The wound area was quantified using imageJ software. Data are means ± S.D of two independent experiments. *P <0.05 compared to the control.

Since *GABARAPL1* has been previously described as a tumor suppressor gene [47, 48], we wondered whether the role of GABARAPL1 in cancer was dependent of its conjugation to autophagosomes. To answer this question, we examined the effect of GABARAPL1 or GABARAPL1 G116A overexpression on cancer cell phenotypes. As previously shown, overexpression of GABARAPL1 led to a decrease in cell proliferation [47]. However, overexpression of GABARAPL1 G116A led to cell proliferation rates similar to those obtained in control cells (Figure 5A). This conclusion was the same regarding other *in vitro* cancer cell phenotypes such as clonogenicity, cell adhesion, anchorage independent-cell proliferation and invasion (Figures 5B-5E). However, different results were obtained regarding cell migration. Indeed, overexpression of GABARAPL1 decreased cell migration in a transwell assay, but surprisingly, overexpression of GABARAPL1 G116A also led to a decrease in cell migration (Figure 5F). In order to confirm these results, we studied cell migration using a wound healing assay. This experiment led to similar results suggesting that overexpression of GABARAPL1 and GABARAPL1 G116A decreased migration rates (Figure 5G). We next wanted to confirm the effects of GABARAPL1 and GABARAPL1 G116A on cell cancer phenotype in a second breast cancer cell lines. To do so, the luminal BT474 breast cancer cell lines was first subjected to transient transfection with the Ctrl, the Flag:GABARAPL1:6His (GABARAPL1) or the Flag:GABARAPL1-G116A:6His (G116A)-expressing vectors (Supplementary Figure S4A) and then analyzed using proliferation and migration assays (Supplementary Figure S4B and C). As already observed for cell migration in the MCF-7 cells, overexpression of GABARAPL1 and GABARAPL1 G116A in BT474 cells led to a decrease in cell proliferation and migration. These results suggested that GABARAPL1 conjugation to autophagosomes may not be necessary for its role in cancer cell regulation *in vitro*.

**Figure 6:**
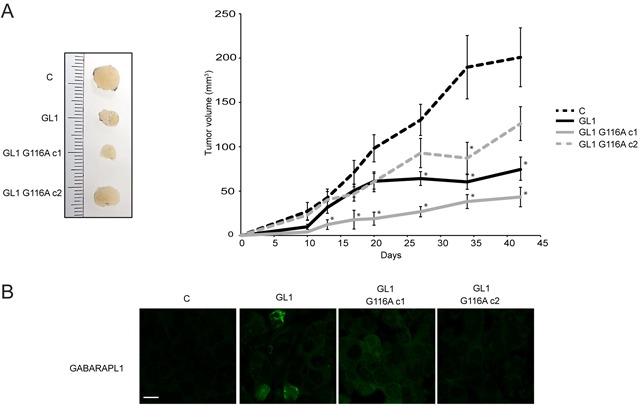
The G116A mutation did not modify the effect of GABARAPL1 on tumor growth *in vivo* Growth of MCF-7 C, GABARAPL1 and GABARAPL1 G116A cells injected subcutaneously in Rag γ/c mice (n = 12 per group). One week prior to cell inoculation and until the end of the experiment, estrogen was administrated at 1 μg/ml in drinking water. **A.** 13 days after injection, the tumor volume was measured twice a week and the tumor volume was calculated using the formula: V= ½ (a x b^2^), where a is the longest tumor axis, and b is the shortest tumor axis. 42 days after injection, tumors were fixed in formol and photographed. Data are means ± S.E.M. of three independent experiments, *P <0.05, compared to the control. **B.** Immunofluorescence analysis of GABARAPL1 in tissue sections from tumors fixed in formol. A representative image of 2 independent experiments is shown. Scale bar represents 10 μm.

In order to conclude about the effect of GABARAPL1 and GABARAPL1 G116A in cancer, we studied *in vivo* the tumor growth after injection of MCF-7 C, GABARAPL1 or GABARAPL1 G116A cells into Rag γ/c mice. The tumor size was monitored for two months following injection. According to previously published data [50], overexpression of GABARAPL1 decreased the size and inhibited the growth of the tumors *in vivo* (Figure 6A). Overexpression of GABARAPL1 G116A also led to a significant decrease in tumor growth. In order to confirm the overexpression of GABARAPL1 and GABARAPL1 G116A in the respective tumor, we next wanted to analyze levels of these proteins in tumors by IHC (Figure 6B). MCF-7 GABARAPL1 G116A c1 cells showed a GABARAPL1 staining similar to those observed in MCF-7 GABARAPL1 cells whereas MCF-7 GABARAPL1 G116A c2 cells presented a lower GABARAPL1 staining. These results were consistent with those described *in vitro* by western blotting (Figure 1B). Interestingly, we noted that GABARAPL1 expression levels seemed to be inversely correlated with tumor growth, but we cannot exclude that there are other intrinsic differences which may influence tumor growth unrelated to GABARAPL1 expression. Moreover, only MCF-7 GABARAPL1 cells showed a GABARAPL1 vesicular staining in tissue sections from tumors. These results suggested that GABARAPL1 could inhibit tumor growth independently of its conjugation to autophagosomes.

## DISCUSSION

GABARAPL1 belongs to the ATG8 family whose function in autophagy has been far less studied than the one linked to the members of the LC3 family. During autophagy, GABARAPL1 is associated with autophagic vesicles and acts with other GABARAP family members to regulate autophagosome maturation [23, 24] and autophagosome-lysosome fusion [56]. Moreover, several studies have highlighted a role of GABARAPL1 as a tumor suppressor protein [25, 47, 50]. This tumor suppressive function has been suggested to be dependent on selective autophagy degradation and its interaction with SQSTM1 [50].

**Figure 7:**
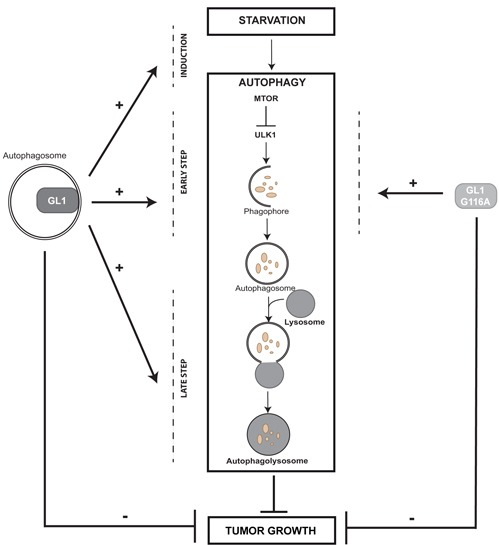
GABARAPL1 functions in autophagy and cancer Our results demonstrated that GABARAPL1, which is linked to the autophagosome, enhances basal and induced autophagy. GABARAPL1 also increases early stages of autophagy through regulation of MTOR or ULK1 activity and late stages of this process through regulation of lysosome activity and autophagosome/lysosome fusion. GABARAPL1 G116A, which is not linked to the autophagosome, can only enhance basal autophagy through regulation of the early stages of this process. However, GABARAPL1 and GABARAPL1 G116A both inhibit tumor growth *in vivo* suggesting that GABARAPL1 conjugation to autophagosomes as well as its functions during late stages of autophagy is not required for its tumor suppressive functions.

In this study, we demonstrated that GABARAPL1 plays a major role during late stages of autophagy, but can regulate earlier steps of autophagy, as well. More importantly, we showed that these two functions do not necessarily require the conjugation of GABARAPL1 to autophagosomes and significantly, we demonstrated that the tumor suppressive function of GABARAPL1 seems to be independent of its conjugation to autophagosomes (see model in Figure 7).

To investigate the role of GABARAPL1 conjugation to autophagosomes on its tumor suppressive function, we designed two *in vitro* models: one MCF-7 cell line stably overexpressing GABARAPL1 and two stably overexpressing the GABARAPL1 G116A mutant protein which is no longer able to be linked onto phospholipids [23]. In our models, the G116A mutation indeed impaired the cleavage, and subsequently the lipidation and conjugaison of GABARAPL1 to autophagosomes (Figures 1D-1E). Our data are consistent with previous studies describing similar results for the G120A mutation in LC3 or the G116A mutation in GABARAP or GABARAPL2/GATE-16 [19, 21, 53]. Nevertheless, these previous studies have only investigated the requirement for the C-terminal processing of GABARAP family members on their function in intracellular transport.

GABARAPL1, like the other members of the ATG8 family, can interact with SQSTM1 to enhance the degradation of ubiquitinylated aggregates through selective autophagy [29]. In our models, the G116A mutation impaired the interaction of GABARAPL1 with SQSTM1 (Figure 2E), suggesting that this protein only interacts with GABARAPL1 when it is localized at the surface of the autophagosomes and that the processing of GABARAPL1 is required for its function during selective autophagy with SQSTM1. These results are consistent with data demonstrating that an ATG4B mutant or the mutant LC3 G120A, which both impaired the maturation and lipidation of LC3, inhibited the interaction of LC3 with SQSTM1; suggesting that SQSTM1 preferentially interacted with LC3-II, the lipidated form of LC3, to be efficiently recruited to the autophagosomes and transported into autophagolysosomes [57, 58]. Interestingly, it has been also demonstrated that the mutation of the LC3-binding site in SQSTM1 inhibited the SQSTM1-LC3 interaction and inhibited the degradation of specific substrates through selective autophagy without affecting overall autophagy [59]. Our data are consistent with these results and confirm that the processing of GABARAPL1, like those of other ATG8 family members, could be important for its conjugation to autophagosomes, its interaction with SQSTM1 and its function in selective autophagy.

Previously, we have demonstrated that a knock-down of GABARAPL1 led to a decrease in basal autophagic flux which was suggested to be linked to a decrease in lysosome number [25]. As expected, overexpression of GABARAPL1 in MCF-7 led to an increase in basal autophagic flux. Surprisingly, overexpression of the GABARAPL1 G116A mutant led to a similar result suggesting that the function of GABARAPL1 in basal autophagy might be independent of its conjugation to autophagosomes (Figures 2B and 2D). We have shown that GABARAPL1 and GABARAPL1 G116A both reduced MTOR activation, inhibited ULK1 phosphorylation (Ser757) mediated by MTOR and increased ULK1 phosphorylation (Ser555) mediated by AMPK during the early steps of autophagy (Figure 4). It has been described that ULK1 inhibits MTOR through the phosphorylation of RAPTOR inducing a positive regulatory loop of autophagy induction [60]. We therefore suggested that GABARAPL1, through its interaction with ULK1 [26, 27], could activate this protein leading to the subsequent inhibition of MTOR. In a recent study, Tooze and colleagues have shown that, in HEK293 cells, GABARAP knock-down decreases ULK1 activation demonstrated by the reduction of the phosphorylation in one of its target, ATG13. They also showed that GABARAPL1 interacts with ULK1 *via* its LIR motif but independently of its lipidation [61]. Moreover, it has also been shown in our laboratory, that GABARAPL1 can interact with the energy sensor AMPK (unpublished data), which has been previously shown to inhibit MTOR and activate ULK1 by phosphorylation (Ser555) [5, 8, 62]. Our results are therefore in agreement with previous studies suggesting a link between basal autophagy and MTOR activity [63]. One hypothesis would be that, in fed condition, GABARAPL1 could recruit AMPK in close proximity of the ULK1 complex leading to the increased ULK1 activation, decreased MTOR activity and the induction of basal autophagy.

During induced autophagy following EBSS treatment, overexpression of GABARAPL1, but not GABARAPL1 G116A, led to an increase in autophagic flux suggesting that the role of GABARAPL1 during induced autophagy requires its conjugation to autophagosomes (Figures 2C-2D). This function could be associated with its role in late stages of autophagy. Indeed, overexpression of GABARAPL1, but not GABARAPL1 G116A, led to an increased lysosomal activity and autophagosome/lysosome fusion (Figure 3). These results are in agreement with the recent literature which identified GABARAP family members as primary contributors to starvation-induced autophagy by the regulation of autophagosome-lysosome fusion [56]. Recently, it has been demonstrated that ATG8 family members, localized on the surface of the autophagosomes, could favor autophagosome/lysosome fusion through their interaction with the protein PLEKHM1 (pleckstrin homology domain containing, family M [with RUN domain] member 1) at the lysosomal surface [64, 65]. It has been also demonstrated that the members of the GABARAP family can recruit PI4KIIα (Phosphatidylinositol 4-kinase IIα) to the autophagosomes leading to the generation of PI4P (Phosphatidylinositol 4-phosphate) which favor autophagosome/lysosome fusion [66]. These observations could explain the increase in lysosome activity and autophagosome/lysosome fusion observed in the presence of the membrane-bound GABARAPL1 but not the cytosolic GABARAPL1 G116A. Moreover, GABARAPL1 is known to interact with microtubules and favor their polymerization [14], and microtubules have been shown to regulate autophagosome/lysosome fusion by relocalizing these organelles to the juxta-nuclear region during autophagy [67, 68]. We could then hypothesize that GABARAPL1, but not GABARAPL1 G116A, could improve microtubule polymerization and therefore facilitate the transport of autophagosomes and lysosomes to the juxta-nuclear region to lead to increased autophagosome/lysosome fusion. Moreover, during autophagy, lysosome activation has been described to require two mechanisms: MTOR inhibition and autophagosome/lysosome fusion [54]. In fact, MTOR not only inhibits ULK1 during early stages of autophagy but also reduces late stages of autophagy through the inhibition of lysosomal function. These data could explain the increased lysosome activity observed in GABARAPL1 overexpressing cells which might present both MTOR inhibition and increased autophagosome/lysosome fusion whereas GABARAPL1 G116A overexpressing cells would only display MTOR inhibition.

Altogether, our results therefore suggest that GABARAPL1 could act during early and late steps of autophagy, functions which are either dependent or independent of its conjugation to autophagosomes. Since GABARAPL1 has also been described as a tumor suppressor protein in breast cancer, we then wondered whether the tumor suppressive function of GABARAPL1 require its conjugation to autophagosomes. *In vitro*, overexpression of GABARAPL1 led to an inhibition of cancer cell phenotypes (proliferation, clonogenicity, adhesion and invasion) which confirmed previous data obtained in the laboratory (Figures 5A-5E) [25, 47]. Interestingly, the G116A mutation inhibited GABARAPL1 tumor suppressive function on several phenotypes apart from migration in MCF7 cells *in vitro* (Figures 5F-5G). Furthermore, the G116A mutation did not alter the effect of GABARAPL1 on cell proliferation and migration phenotypes in another breast cancer cell line BT474 *in vitro* (Figures S4). These data therefore suggest that GABARAPL1 conjugation to autophagosomes may not be necessary for its function in cancer cells *in vitro*.

In order to characterize the effect of GABARAPL1 conjugation to autophagosomes on cancer progression *in vivo*, we studied tumor growth following injection of GABARAPL1 and GABARAPL1 G116A-overexpressing cells into Rag γ/c mice (Figure 6A). Overexpression of GABARAPL1 led to a decrease in tumor size confirming previous results obtained in *nude* mice [50]. Overexpression of GABARAPL1 G116A also led to a decrease in tumor growth suggesting that GABARAPL1 conjugation to autophagosomes could not be necessary for its tumor suppressive function *in vitro* as well as *in vivo*. Moreover, given that GABARAPL1 G116A, which predominately cannot conjugate to autophagosomes and interacts less with SQSTM1, still presented a tumor suppressor role, we might think that GABARAPL1 function in selective autophagy is not essential for its tumor suppressive function. GABARAPL1 and GABARAPL1 G116A could present a tumor suppressor role through the inhibition of MTOR which has been previously shown to be involved in cell proliferation and tumor progression [69–71]. But, we cannot exclude that GABARAPL1 might present a tumor suppressor role through functions independent of its role in autophagy. For example, BECN1, which is a protein involved in the initiation of autophagy and previously described as a tumor suppressor gene frequently deleted or downregulated in cancer [42, 72, 73], can inhibit tumorigenesis through the inhibition of MCL-1 stabilization or WNT1 (wingless-type MMTV integration site family, member 1) activation, two mechanisms independent of autophagy [74, 75].

Our results demonstrate that GABARAPL1 can act during early and late steps of autophagy, independently or not of its conjugation to autophagosomes, respectively. We also showed that its conjugation to autophagosomes as well as its function during late stages of autophagy and selective autophagy is probably not required for its tumor suppressive function *in vivo* (Figure 7).

## MATERIALS AND METHODS

### Reagents and antibodies

Earle's balanced salt solution (EBSS, E3024), bafilomycin A1 (B1793), chloroquine (C6628), MG132 (C2211) and NH_4_Cl (A0171) were purchased from Sigma-Aldrich. For the western blotting experiments, the following antibodies were used: polyclonal anti-GABARAPL1 (Proteintech, 11010-1-AP, 1:1000), monoclonal anti-GABARAPL1/GABARAP (Millipore, AB15278, 1:1000), polyclonal anti-LC3 (Sigma-Aldrich, L8918, 1:3000), monoclonal anti-LAMP1 (Abcam, Ab25630, 1:1000), monoclonal anti-MTOR (Cell signaling, #2983, 1:1000), polyclonal anti-phospho-MTOR (Cell signaling, #2974, 1:1000), polyclonal anti-P70S6K (Cell signaling, #9202, 1:1000), polyclonal anti-phospho-P70S6K (Cell signaling, #9205, 1:1000), monoclonal anti-phospho-ULK1 (Ser555) (Cell signaling, #5869, 1:1000), monoclonal anti-phospho-ULK1 (Ser757) (Cell signaling, #14202, 1:1000), monoclonal anti-ULK1 (Cell signaling, #8054, 1:1000), polyclonal anti-GFP (Chemicon Millipore, AB3080, 1:1000), polyclonal anti-ACTIN (Sigma-Aldrich, A5060, 1:15000), polyclonal anti-rabbit (P.A.R.I.S, BI2407, 1:10000) and polyclonal anti-mouse (P.A.R.I.S, BI24130, 1:10000). For the immunofluorescence and P-LISA experiments, the following antibodies were used: polyclonal anti-GABARAPL1 (Proteintech, 11010-1-AP, 1:200), monoclonal anti-LAMP1 (Abcam, Ab25630, 1:100), monoclonal anti-SQSTM1 (Santa Cruz, sc-28359, 1:250), Alexa Fluor 555 goat anti-mouse (Life technologies, A-21422, 1:800), Alexa Fluor 488 goat anti-rabbit (Life technologies, A-11008, 1:800) and polyclonal anti-rabbit FITC (P.A.R.I.S, BI2107, 1:200).

### Cell culture and treatment

MCF-7 control cells (C) and MCF-7 Flag:GABARAPL1:6His (GABARAPL1) cells were obtained previously in our laboratory following transfection with pcDNA3.1 control, pcDNA3.1-Flag-GABARAPL1-(His)6 [47]. MCF-7-Flag:GABARAPL1-G116A:6his (GABARAPL1 G116A c1 and c2) cells were obtained in the same way following transfection with pcDNA3.1-Flag-GABARAPL1-G116A-(His)6 vectors. The cells were cultured in Dulbecco's minimum essential medium (DMEM) (PAA, E15-891) supplemented with 100 μg/ml penicillin/streptomycin (PAA, P11-010) and 10% fetal bovine serum (FBS) (PAA, A15-101) in a 5 % CO_2_ incubator at 37°C. To inhibit autophagosome/lysosome fusion, cells were incubated for 2 h in complete medium supplemented with 100 nM bafilomycin A1, 40 μM chloroquine or 50 mM NH_4_Cl. To induce autophagy, cells were incubated in EBSS for 4h at 37°C. To inhibit proteasome degradation, cells were cultured in complete medium supplemented with 2 μM MG132 for 16 h.

For transient transfection, 2 μg of pGFP, pGABARAPL1-GFP, pGABARAPL1-G116A-GFP, 200 μl Jetprime Buffer and 4 μl Jetprime reagent (Polyplus transfection, 114-07) were used per reaction according to the manufacturer's protocol.

### Western blotting

Cells were scraped, harvested and lysed in SB1X. Protein lysates were sonicated for 5 s before loading (Sonics and Materials), separated on a 10 or 12.5 % sodium dodecyl sulfate-polyacrylamide gel electrophoresis (SDS-PAGE) before being transferred onto a polyvinylidene difluoride (PVDF) membrane (Bio-Rad, 162-0177). The membrane was blocked with 5 % nonfat milk in Tris-buffered saline with Tween 20 (TBS-T) (20 mM Tris-HCl, pH 7.6, 137 mM NaCl, 0.1 % Tween 20) and incubated with primary antibodies at the previously indicated dilutions. Immunoreactive bands were detected using secondary goat horseradish peroxidase (HRP)-coupled anti-mouse or anti-rabbit antibodies and the *p*-coumaric acid-enhanced chemiluminescent (PCA-ECL) solution [76] and were analyzed using the ChemiDoc XRS+ system (Biorad). Protein levels were quantified using the Image Lab software.

### RT-PCR

Total RNAs were extracted as previously described [77]. For RT-PCR analysis, 2 μg of total RNAs were reverse transcribed using the RevertAid M-MulV Reverse Transcriptase to obtain cDNA (Sigma, M1302). The exogenous *GABARAPL1* primer sequences (Flag:GABARAPL1:6His) were: T7 sens 5′-TAAATACGACTCACTATAGGG-3′ and BamH1 reverse 5′-CGCGGATCCGCCTTTCCCATAGACACTCTC-3′ and the following primer sequences were used for *GAPDH*: GAPDH S 5′-GCGAGATCCCTCCAAAATCA-3′ and GAPDH R 5′-TGTGGTCATGAGTCCTTCCA-3′. The DreamTaq DNA polymerase (Life technologies, EP0711) was used to amplify *GABARAPL1* and *GAPDH* from the template cDNA. *GAPDH* was used as an internal control. Polymerase chain reaction was performed for 35 cycles: initial denaturation at 94°C for 5 min, denaturation at 94°C for 30 seconds, annealing at 52°C for 30 seconds, extension at 72°C for 1 min and final extension at 72°C for 10 min. All PCR products were analysed by electrophoresis on a 2% agarose gel and signals were visualized using a Gel doc EZ imager (Biorad).

### Immunofluorescence and confocal microscopy

MCF-7 cells were plated on coverslips in 24-well plates at a density of 10^5^ cells/well. For transient transfection, the GFP-LC3 (green fluorescent protein-microtubule-associated protein light chain 3) plasmid was kindly provided by Dr. Elazar (Weizmann Institute, Israël) and the ptf-LC3 vector was purchased from Addgene (137624). Plasmids were transfected using the Jetprime reagent (Polyplus transfection, 114-07) according to the manufacturer's protocol. After the designated treatments, cells were washed with phosphate-buffered saline (PBS), fixed with 4 % PFA (paraformaldehyde) in PBS for 15 min at room temperature and mounted in Vectashield Hardset (Vector laboratories, H1400). The cells were then examined and photographed using a confocal microscope (Olympus Fluoview FV1000). For ptf-LC3, green, red and yellow puncta were counted using the “Green and Red puncta colocalization tool” designed for ImageJ. For each experiment, 20 cells were randomly selected.

For Lysotracker staining, cells duplicate were stained with 500 nM of LysoTracker® Red DND-99 (Life technologies, L-7528) for 1 h. Cells were then washed with PBS, fixed with 4 % PFA in PBS for 15 min at room temperature and mounted with Vectashield Hardset. The cells were then examined and photographed using a confocal microscope (Olympus Fluoview FV1000). For each group, 100 cells were randomly selected.

For GABARAPL1, LAMP1 and SQSTM1 immunostaining, cells were washed with PBS and fixed with 4 % PFA in PBS for 15 min at room temperature. Cells were then permeabilized with 0.2 % Triton-X100 in PBS for 5 min, washed with PBS, blocked with 5 % BSA (bovine serum albumin) in PBS for 45 min, incubated with primary antibodies overnight at 4°C, at the previously indicated dilutions, and finally with a secondary goat anti-rabbit-FITC or an Alexa Fluor 555 goat anti-mouse for 1 h. The cells were then mounted with Vectashield Hardset and analyzed using a confocal microscope. Each picture is representative of a typical cell staining observed in 10 fields chosen at random. Colocalization of the autophagosome marker GFP-LC3 and the lysosomal marker LAMP1 was analyzed using a confocal microscope and the Pearson's coefficient using coloc_2 (ImageJ software). For each group, 25 cells were randomly selected.

For immunofluorescence staining of tumor tissue, tumors were embeded in Tissue-Tek (Microm microtek, F/62550-1) and sliced using a Cryostat (Microm microtech, HM560) (Plateau technique d’histopatholigie, DImaCell platform). The slides were incubated at 95°C for 40 min in sodium citrate buffer (10 mM sodium citrate, pH 6), overnight with the previously described GABARAPL1 antibody and 1 h at room temperature with an Alexa Fluor 488 goat anti-rabbit secondary antibody at previously indicated dilutions. After each incubation, the slides were rinsed thrice in 1% PBS-Triton X-100. After being mounted in PBS-glycerol mounting medium, the slides were observed and analyzed using a confocal microscope (Olympus Fluoview FV1000). Two independent experiments were performed.

### Cell migration and invasion assays

10^5^ cells were diluted in 250 μl serum-free medium, added to the upper chamber of the Boyden modified chamber™ (SPL Life Sciences) and incubated for 24 h at 37°C. For the cell invasion assay, 50 μl of extra cellular matrix (ECM) gel (Sigma-Aldrich, E1270, 1 mg/ml) were added to the upper chamber 5 h before cell seeding. The invasive cells were fixed, stained with 2 % crystal violet and images of each membrane were acquired. Finally, the invasive cells located in the lower chamber were counted manually. 10 FOV were randomly selected and the number of invasive and migrative cells was determined. Three independent experiments were performed in duplicate.

### Cell proliferation

Cells were plated in 96-well plates at a density of 1.5 × 10^3^ cells/well and cell proliferation experiments were conducted over a 7-day period using MTT [3-(4,5-dimethylthiazol-2-yl)-2,5- diphenyl tetrazolium bromide] (Sigma-Aldrich, M2128). Each day, 100 μl of 100 mM MTT solution in Hank's were added to the cells after the removal of the supernatant. After a 2 h incubation, the formazan crystals were dissolved in 50 μl of DMSO (dimethyl sulfoxide) (Euromedex, UD8050-A) and the absorbance was quantified at 549 nm using a microplate reader (Multiskan FC, ThermoScientific). For each clone, three independent experiments were performed in 24 wells of a 96 well-plate.

### Colony formation assay

Cells were plated duplicate in 6-well plates at a density of 2 × 10^3^ cells/well. After 15 days, colonies were fixed with 100% ethanol for 10 min and stained with 2% crystal violet for 10 min. The dye in excess was rinced with water and the colony number was imaged using a ChemiDoc XRS+ and Image Lab 2.0 software (Biorad). Quantitative changes in clonogenicity were determined by counting the colonies, using Bio-Rad Vision-Capt software. Three independent experiments were performed in duplicate.

### Cell-matrix adhesion assay

Cells were plated in 24-well plates at a density of 4 × 10^5^ cells/well in serum-free DMEM for 1 h and 3 h at 37°C (three wells/cell line: one well to control the number of seeded cells and the two others for the two different times after seeding). After washing, adherent cells were collected and pelleted. Then, cells were counted using a Malassez cell. Results were expressed as the ratio of adherent cells versus total seeded cells. Three independent experiments were performed in triplicate.

### Anchorage independent cell proliferation

Cells were seeded in 6-well plates at a density of 5 × 10^5^ cells/well in 0.3% agar (Fisher Scientific, 10776644). Before seeding, 0.6% agar was added into each well. The cell layers were then covered with complete medium and cultured for 25 days at 37°C. Images from four representative fields of each well were taken and analyzed. Three independent experiments were performed in duplicate.

### Wound healing assay

Cells were seeded in 6-well plates at a density of 5 × 10^5^ cells/well. Following confluency, two artificial wound per well were performed into the monolayers using a micropipette tip. After wounding, the tissue culture medium was removed, and cells were washed at least twice in PBS to eliminate detached cells. Wound closure was monitored after 24 h, 48 h and 72 h using image J software. The migration of cells was expressed as the percentage of wound closure: % of wound closure = [(A_t = 0 h_ - A_t = ∆h_)/A_t = 0 h_] ×100%. A_t = 0h_ is the wound area measured immediately after scratching, and A_t = Δh_ is the wound area measured at 24 h, 48 h and 72 h after scratching. Two independent experiments were performed in 8 replicates.

### Xenograft experiments

Rag γ/c mice were obtained from Taconic (Germantown, NY, USA) and maintained in the UMR1098 animal facility (agreement number #C25-056-7). Approval for animal experimentation and care was received from the “Services Vétérinaires de la Santé et de la Protection Animale” delivered by the “Ministère de l’Agriculture”, Paris, France and experimental procedures were approved by a local ethic committee. One week prior to cell inoculation and until the end of the experiment, estrogens were administrated at 1 μg/ml in drinking water. A total of 1.5 × 10^6^ cells of the different cell lines resuspended in 100 μL of PBS were inoculated subcutaneously in RAGg/c mice (n = 9 per group) and tumor growth was monitored two times a week in each group. Tumor volume was calculated by the formula V= ½ (a x b^2^), where a is the longest tumor axis and b is the shortest tumor axis. When tumors reached 1 cm in diameter, mice were sacrified and each tumor was fixed in formol and photographed.

### Proximity ligation *in situ* assay (P-LISA)

MCF-7 cells were cultured for 24 h on coverslips and then fixed and permeabilized using cold methanol for 20 min at −20°C. P-LISA staining was performed according to the OlinkBioscience's recommendations using Duolink® In Situ Detection Reagents Red kit (Sigma-Aldrich, DUO92008) and as previously described [51]. The number of red dots and the intensity per dots were counted using the Blobfinder software. 200 cells were randomly selected in 5 fields.

### Transmission Electron microscopy

MCF-7 cells were washed in 0.1M Sörensen buffer, pH 7.3. Then, cells were fixed with 2% glutaraldehyde (Sigma Aldrich, G5882) in 0.1M Sörensen buffer, pH 7.3 for 1 h at 4°C, and post-fixed in the mixture 1% osmium tetroxide (Sigma Aldrich, 75632) and 1.5% potassium ferricyanide (Sigma Aldrich, 702587) in 0.2M Sörensen buffer, pH 7.3 for 1 h at 4°C. The cells cultures were dehydrated through a graded series of ethanol to 100%, embedded in Poly/Bed 812 resin (Polysciences, 21844-1) and polymerized for 48h at 60°C. Ultrathin sections were collected on 100 mesh nickel grids coated formvar-carbon (Delta microscopy), stained with uranyl acetate (Polysciences, 21447-25) and lead citrate. Then, they were imaged using a Technaï 12 Biotwin TEM microscope (Primacen Platform, University of Rouen). Representative image of 60 pictures for each cell lines is shown.

### Flow cytometry

Cells were plated in 24-well plates at a concentration of 2 × 10^5^ cells/well and incubated at 37°C overnight following the different treatments. Cells were stained with MagicRed (Immunochemistry technologies) according to the manufacturer's protocol or 50 nM LysoTracker® Green DND-26 (Life technologies, L-7526) for 45 min. After two PBS washes, cells were then trypsinized and resuspended with 500 μl complete medium before being harvested at 5,000 g for 5 min and resuspended in 250 μl PBS. Cells (10,000 events) were then examined using a FC500 Beckman Coulter flow cytometer. Data were acquired and analyzed using the Flowing software.

### Statistical analysis

Statistical analyses were carried out using a Student's t test. A p value <0.05 was considered as statistically significant.

## SUPPLEMENTARY FIGURES


