# Supplementary material for: GABARAPL1 tumor suppressive function is independent of its conjugation to autophagosomes in MCF-7 breast cancer cells
Source: Oncotarget. 2017 Jul 27;8(34):55998–6020. doi: 10.18632/oncotarget.19639 (PMC5593540; doi:10.18632/oncotarget.19639)
Supplement: Supplementary file 1 [file oncotarget-08-55998-s001.pdf]

GABARAPL1 tumor suppressive function is independent of its conjugation to autophagosomes in MCF-7 breast cancer cells

SUPPLEMENTARY MATERIALS

BT474 cell culture and proliferation and migration assays

BT474 control cells (pCtrl), BT474 Flag:GABARAPL1:6His (pGL1) and BT474 Flag:GABARAPL1-G116A:6his (pG116A) cells were obtained following transient transfection with pcDNA3.1, pcDNA3.1-Flag-GABARAPL1-(His)6 and pcDNA3.1-Flag-GABARAPL1-G116A-(His)6 vectors, respectively. Transient transfections were performed using 15 µg of each vector and 45 µl TransIT®-2020 Transfection reagent (Euromedex, MIR5400) per reaction, according to the manufacturer's protocol. The cells were cultured in Roswell Park Memorial Institute medium (RPMI, Dustcher, 702519) supplemented with 100 µg/ml penicillin/streptomycin (PAA, P11-010) and 10% fetal bovine serum (FBS) (PAA, A15-101) in a 5 % CO_2 incubator at 37°C.

The protocols used for cell migration (4 x 10^4 cells) and proliferation (3 x 10^3 cells/well) assays are the one previously described for the MCF-7 cells. For the cell migration assay, three independent experiments were performed in duplicate. For the proliferation assay, three independent experiments were performed in 8 wells of a 96 well-plate.

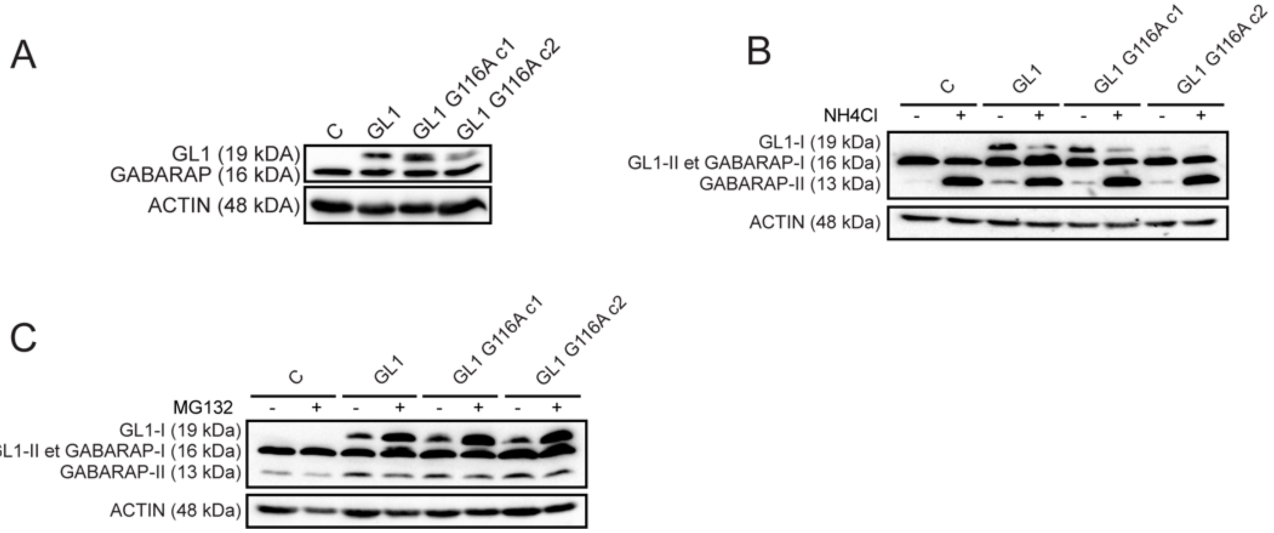

Supplementary Figure 1: Effect of GABARAPL1 and GABARAPL1 G116A overexpression on GABARAP expression and degradation. **A**. Western blotting analysis of GABARAP in MCF-7 C, GABARAPL1 and GABARAPL1 G116A cells using anti-GABARAPL1/GABARAP antibodies. Data representative of three independent experiments performed are shown. **B**. Western blotting analysis of GABARAP in MCF-7 C, GABARAPL1 and GABARAPL1 G116A cells cultured in medium with or without 50 mM NH_4Cl for 2h. Data representative of three independent experiments performed are shown. **C**. Western blotting analysis of GABARAP in MCF-7 C, GABARAPL1 and GABARAPL1 G116A cells cultured in medium with or without 2 µM MG132 for 16h. Data representative of two independent experiments performed are shown.

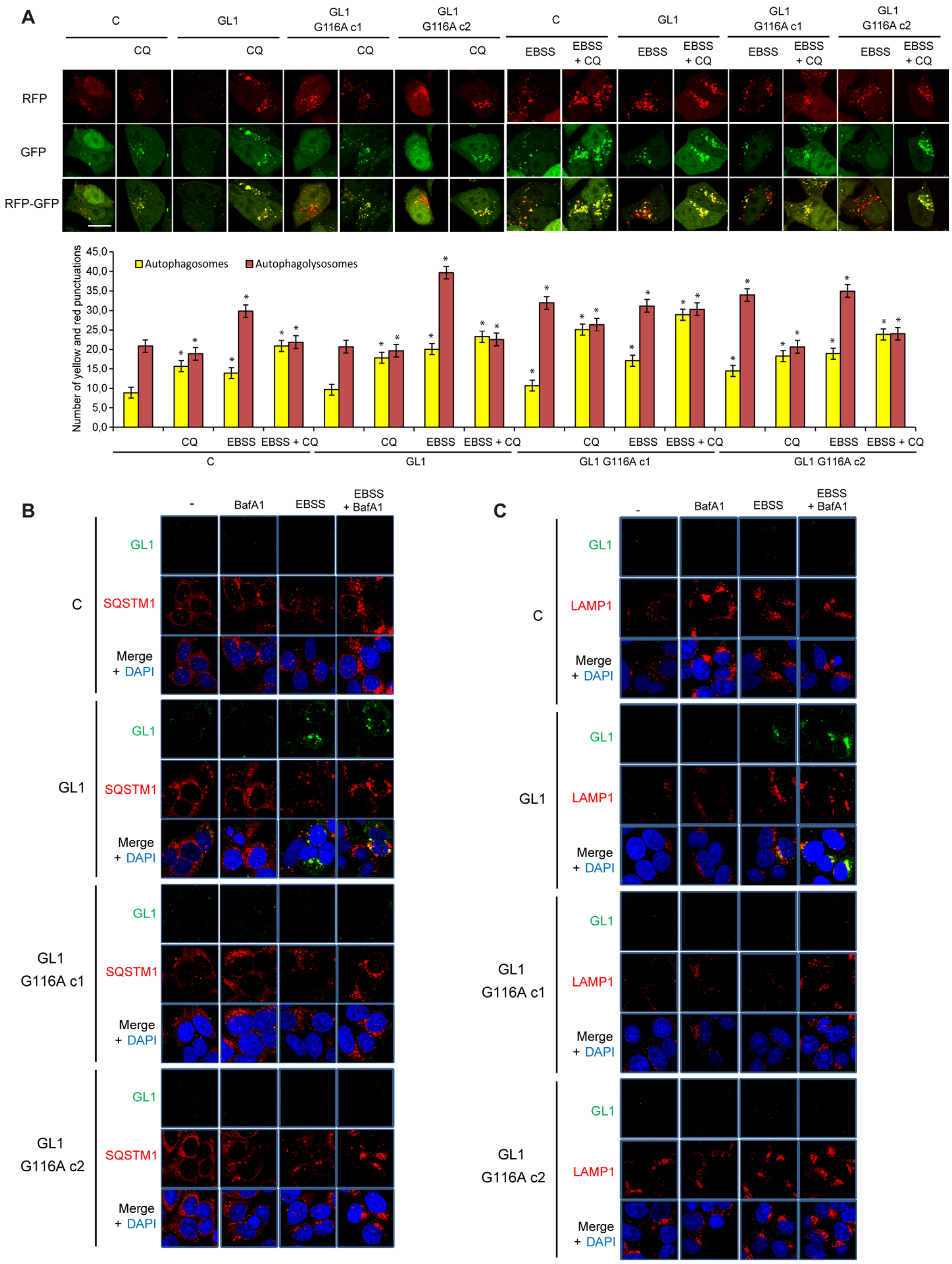

Supplementary Figure 2: The G116A mutation impaired the effect of GABARAPL1 on induced but not basal autophagy. A. GFP-RFP-LC3 puncta analysis in MCF-7 C, GABARAPL1 and GABARAPL1 G116A cells transfected with the ptf-LC3 vector and cultured in medium or EBSS with or without 40 µM CQ. Each picture is representative of a typical cell staining observed in 20 fields chosen at random. Red and yellow puncta were counted using the ImageJ software (Green and Red puncta colocalization tool). For each experiment, 20 cells were randomly selected. Data are means ± S.E.M. of two independent experiments, *P <0.05, yellow and red puncta compared to the respective yellow or red puncta of control cells. Scale bar represents 10 µm. **B**. Colocalization of SQSTM1 and GABARAPL1 in MCF-7 C, GABARAPL1 and GABARAPL1 G116A cells cultured in medium or EBSS for 4h with or without 100 nM BafA1 for 2h. A representative image of three independent experiments is shown. C. Colocalization of LAMP1 and GABARAPL1 in MCF-7 **C**, GABARAPL1 and GABARAPL1 G116A cells cultured in medium or EBSS for 4h with or without 100 nM BafA1 for 2h. A representative image of three independent experiments is shown.

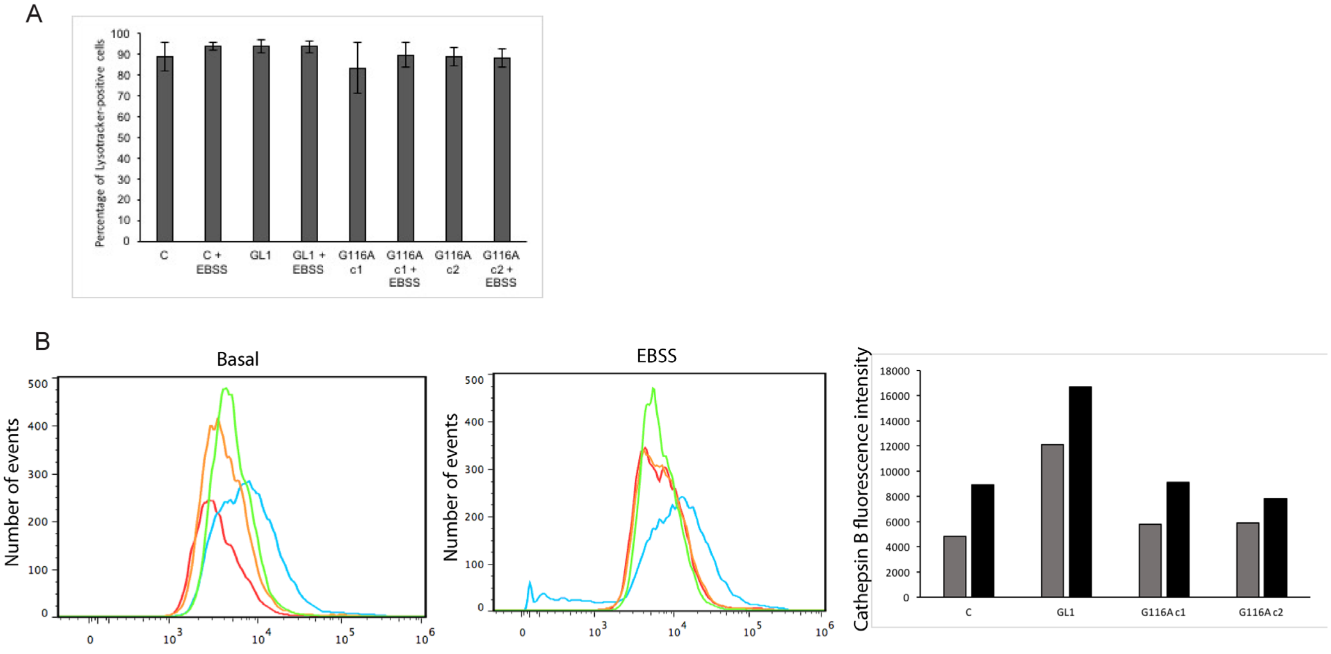

Supplementary Figure 3: GABARAPL1 and GABARAPL1 G116A expression did not modify percentage of Lysotracker-positive cells. A. Lysotracker staining was analyzed in MCF-7 C, GABARAPL1 and GABARAPL1 G116A cells cultured in medium or EBSS for 4h. Percentage of Lysotracker-positive cells quantified with the Blobfinder software. Data are means ± S.E.M. of three independent experiments performed in duplicate. *P <0.05 compared to the control. **B**. MagicRed fluorescence analysis in MCF-7 C, GABARAPL1 and GABARAPL1 G116A cells cultured in medium or EBSS for 4h using the flowing software. The number of events (left) and quantification data (right) were shown. Intensity of MagicRed fluorescence of one experiment was analyzed by flow cytometry using the Flowing software.

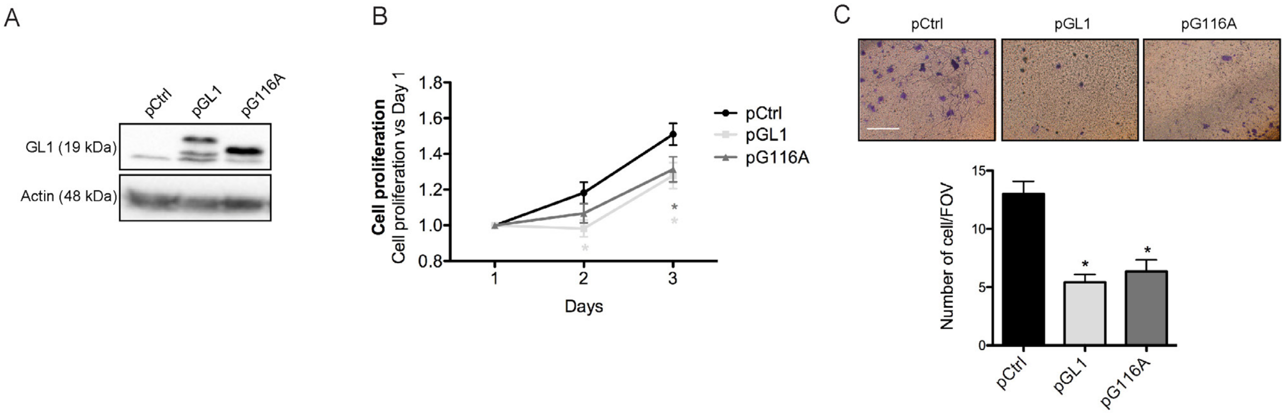

Supplementary Figure 4: The G116A mutation did not alter the effect of GABARAPL1 on proliferation and migration phenotypes in BT474 cells. BT474 cells were transiently transfected with the pCtrl, the pGABARAPL1 or the pGABARAPL1 G116A vector. **A**. Western blotting analysis of GABARAPL1 expression in BT474 pCtrl, BT474 pGABARAPL1 and BT474 pGABARAPL1 G116A cells. Data are representative of three independent experiments. **B**. Growth rate of BT474 pCtrl, BT474 pGABARAPL1 and BT474 pGABARAPL1 G116A cells using MTT assay. Each experiment contains 8 replicates and data are means ± S.E.M of three independent experiments. *P <0.05 compared to the control. **C**. Migration quantification of BT474 pCtrl, BT474 pGABARAPL1 and BT474 pGABARAPL1 G116A cells in Boyden-modified chamber. A representative image of ten fields of view (FOV) of each membrane is shown. These 10 FOV were randomly selected and the number of migrative cells was determined. Each experiment was performed in duplicate and the data are means ± S.E.M of three independent experiments. *P <0.05 compared to the control. The scale bar represents 10 µm.
